# Aphicidal Activity of Surfactants Produced by *Bacillus atrophaeus* L193

**DOI:** 10.3389/fmicb.2018.03114

**Published:** 2018-12-18

**Authors:** Miguel Rodríguez, Ana Marín, Marta Torres, Victoria Béjar, Mercedes Campos, Inmaculada Sampedro

**Affiliations:** ^1^Department of Microbiology, Pharmacy Faculty, Microbial Exopolysaccharide Research Group, Granada, Spain; ^2^Biomedical Research Center (CIBM), Biotechnology Institute, Granada, Spain; ^3^Institute for Integrative Biology of the Cell (I2BC), CEA/CNRS/University of Paris-Sud, University Paris-Saclay, Gif-sur-Yvette, France; ^4^Department of Plant Protection, Estación Experimental del Zaidín, Spanish National Research Council, Granada, Spain

**Keywords:** *Bacillus atrophaeus* L193, biosurfactants, lipopeptides, aphids, insecticidal activity

## Abstract

The biosurfactants produced by *Bacillus atrophaeus* L193 was examined by their use in the control of the aphid *Rhopalosiphum padi* in order to suggest a friendly alternative to chemical pesticides. A screening of different culture media demonstrated the highest biosurfactant production by L193 in TSB supplemented with colloidal chitin. Surfactants, which are produced in large quantities (2.04 g/L), reduce surface tension to 33 mN/m. Electrospray Q-TOFS MS analysis demonstrated that lipopeptides, such as surfactins, fengycins, bacillomycins and iturins, are the predominant metabolites present in biosurfactants produced by strain L193. Treatment with L193 surfactants led to an aphid mortality rate of 59.8% within 24 h. Microscopy analysis showed that these compounds caused insect death by affecting cuticle membranes. An evaluation of aphid feeding activity also demonstrated that aphid feeding capacity is affected by treatment with surfactants. Moreover, microbial cultures of strain L193 and their supernatants also showed high levels of activity against *R. padi*, which is probably due to the presence of surfactants and hydrolytic enzymes such as proteases and glucanases. This study demonstrates that *B. atrophaeus* L193 is an effective treatment for plants affected by aphids.

## Introduction

Aphid species *Rhopalosiphum padi* affects several cereal crops, resulting in severe economic losses in agriculture. These hemiptera insects are considered to be some of the most abundant and economically important aphids affecting both winter and spring wheat crops. *R. padi* leads to plant decline and transmits different phytopathogenic viruses including the barley yellow dwarf virus (BYDV) which causes chlorosis, stunting and yield loss (Biurrun et al., 2010; Liu et al., 2014). It also promotes the growth of fungi, such as *Cladosporium* sp. and *Alternaria* sp. on leaf surfaces through the excretion of sugars, and decreases the rate of plant photosynthesis (Dhami et al., 2013).

Currently, this pest is managed through the application of chemical insecticides. However, this can lead to resistance and pollute subterranean water due to soil lixiviation. In recent years, many predatory insects, though highly inefficient in open fields, have been used to control this pest in greenhouse crops. The use of microorganisms as biocontrol agents, which are typically members of the genus *Bacillus*, is a safe and environmentally friendly alternative to chemical pesticides (Copping, 2004).

Various studies have analysed the efficiency of *Bacillus thuringiensis* in the control of chewing insects such as *Lepidoptera*, *Diptera*, and *Coleoptera* (Bravo et al., 2007; Sanahuja et al., 2011). Nevertheless, few published studies have examined the insecticidal activity of the genus *Bacillus* against sucking insects such as aphids. Thus, studying the potential of these microorganisms as biocontrol agents against aphids could be worthwhile and contribute to the development of environmentally friendly approaches in agriculture.

The objective of this study is to analyse the biopesticidal activity of *Bacillus atrophaeus* L193 against the aphid *R. padi*. Thus, the production and physicochemical characteristics of L193 biosurfactants, as well as their anti-aphid insecticidal activity under controlled conditions were studied. The effect of *B. atrophaeus* L193 surfactant treatment on the aphid membrane was evaluated using microscopy analysis and by studying aphid feeding. These tests were also carried out on *B. atrophaeus* cultures and cell-free supernatants. To the best of our knowledge, this is the first study to evaluate the anti-aphid insecticidal activity of *B. atrophaeus*.

## Materials and Methods

### Microorganism Isolation and Identification

The strain used in this study was isolated from the Malahá salt works (Granada, Spain, 37°06′11.9”N 3°43′14.6”W) after 200 bacteria isolated from several hypersaline environments were screened in order to find the best producers of biosurfactants with insecticidal potential. The methodology used in this screening was the drop collapsing test described by Youssef et al. (2004). The 16S rRNA gene of strain L193 was amplified by PCR and sequenced using universal bacterial primers (Lane, 1991) 16F_B27 (5′AGAGTTTGATCMTGGCTCAG-3′) and 16R_B1488 (5′-CGGTTACCTTGTTAGGACTTCACC-3′). 16S rRNA fragments obtained by PCR were cloned into the pGEM-T vector (Promega^®^) and later transformed into *Escherichia coli* DH5α. Vector plasmids were purified with the aid of an Illustra GFX DNA kit (GE Healthcare^®^) according to the manufacturer’s instructions. Sequencing was carried out in an Illumina NextSeq^TM^ 500 system. The resulting sequences were analysed with the DNAstar Lasergene Seqman programme (Madison, WI, United States). The sequences were identified using the GenBank and EMBL databases (Altschul et al., 1990) and the EzTaxon-e programme (Yoon et al., 2017).

### Biosurfactant Production

Biosurfactant production of L193 strain was tested in the following media: Luria Bertani (LB), LB supplemented with 1% (w/v) colloidal chitin, tryptic soy broth (TSB), TSB supplemented with 1% (w/v) colloidal chitin, Cooper supplemented with 1% (w/v) colloidal chitin, Cooper supplemented with 4% (w/v) glucose and Cooper supplemented with 1% (w/v) colloidal chitin and 4% (w/v) glucose (Cooper et al., 1981). Colloidal chitin was obtained as described by Wu et al. (2009).

To determine biosurfactant production (Song et al., 2013), strain L193 was cultured in 100 mL of each medium as described above at 28°C for 7 days and 130 rpm rotary shaking. The cultures were centrifuged at 13,000 rpm for 20 min, and the cell pellets were freeze-dried. The supernatants were subjected to acid extraction in order to obtain the lipopeptides within it. The supernatant lipopeptides were then freeze-dried, and the difference in yield between cell biomass and biosurfactant was calculated.

### Biosurfactant Detection in L193 Culture on TSB Chitin Medium

In order to detect biosurfactant production on strain L193, the evolution of surface tension in the strain culture was evaluated during 24 h. Additionally, the surfactant activity of the strain L193 culture was studied using the drop-collapsing test, the oil spreading test and the emulsification index. All tests were carried out in triplicate using 1% (v/v) Triton X-100 and sterile water as positive and negative controls, respectively.

Surface tension was measured at 25°C during 24 h using the Wilhelmy plate method (Biswas et al., 2001) with the aid of a Kruss K11 tensiometer. Samples were taken every hour during 24 h from a culture of strain L193 in TSB supplemented with 1% (w/v) colloidal chitin. They were then centrifuged to obtain cell-free supernatants. Three replicates were measured per each sample.

The drop-collapsing test (Youssef et al., 2004) was performed in 96-well micro-titter plates by adding 2 μL mineral oil to each well. After 1 h at room temperature to allow the stabilisation of the oil drops, 5 μL of L193 supernatant were deposited on the mineral oil drop, and the shape of the drop was analysed by visual inspection after 1 min. The reduction in surface tension caused by the presence of biosurfactant in the supernatant produced a flattened oil drop, while a round oil drop was produced in the absence of the biosurfactant.

For the oil-spreading test (Morikawa et al., 2000), 15 μL crude oil were deposited on the surface of a 150-mm diameter Petri dish containing 40 mL destilled water. 10 μL of the biosurfactant were then carefully deposited in the centre of the dish, and the radius of the clearly formed circle was measured in order to calculate the area of oil displacement.

The emulsification index (Cooper and Goldenberg, 1987) of the culture supernatants and the purified biosurfactant were determined in haemolysis tubes by mixing 4 mL of diferent hydrocarbons (such as almond oil, isopropyl mirystate and mineral oil) with an equal volume of cell-free supernatant or biosurfactant. After vortexing at maximum speed for 5 min, the mixtures were left to stabilise at room temperature for 24 h. The emulsification index (E_24_) (%) was expressed as the height of the emulsified fraction in relation to the total height of the mixture.

### Determination of Hydrolytic Enzyme Production

In order to quantify hydrolytic activity, strain L193 was cultured in 100 mL TSB medium supplemented with 1% (w/v) colloidal chitin at 28°C for 5 days in a shaking incubator at 130 rpm. To obtain cell-free supernatants, the cultures were centrifuged at 13,000 rpm for 20 min. Protease, chitinase and amylase activities of the supernatants were tested in 6-mm-diameter wells done in TSB media in Petri dishes containing 50% (v/v) skimmed milk, 1% (w/v) colloidal chitin and 1% (w/v) anhydrous starch, respectively (Barrow and Feltham, 1993). Each activity was tested using 100 μL of the pure supernatant (100% concentration) and the supernatant diluted in water at concentrations of 50% and 25%. For the protease and chitinase determination, plates were incubated at 28°C for 10 days. The radius of the transparent halos around the wells was measured after 5 and 10 days of incubation. Amylase activity was measured after 5 days using a Lugol’s solution (Barrow and Feltham, 1993). A transparent halo arround the wells indicated a positive result for amylase activity, while if the medium remained dark blue/black meant that starch was not hydrolysed, therefore reacting with iodine of the Lugol’s solution and dyeing the medium.

### Genetic Identification of Biosurfactant

Non-ribosomal peptide synthetase (NRPS) genes coding for biosurfactant production were detected by PCR using specific degenerated primers described in Table 1. PCR amplifications were achieved in 50 μL mixtures containing PCR buffer, 2 mM MgCl_2_, 4 mM of each primer, 5U Taq polymerase, 0.2 mM of each dNTP and 80–100 ng of genomic DNA. Amplification conditions were as follows: 95°C for 5 min, 40 cycles at 94°C for 1 min, annealing temperature for 1 min, extension at 72°C for 1 min and a final extension at 72°C for 10 min. Annealing temperatures were 45°C, 43°C, 50°C, and 53°C for Af2/Tf1, As1//Ts2, BmyBF/BmyBR and ItuDF/ItuDR, respectively (Chung et al., 2008; Tapi et al., 2010; Mora et al., 2011). The amplification products were analysed by electrophoresis in a 2% (w/v) agarose gel after staining with RedSafe^TM^ (Intron).

**Table 1:** PCR primers of lipopeptide biosynthesis genes in *Bacillus atrophaeus* L193.

Lipopeptide	Gene	Primers	Primer sequences (5′→3′)	PCR product size (bp)	Reference
Fengycin	*fen*C	Af2 (F)	GAATAYMTCGGMCGTMTKGA	443–455	Tapi et al., 2010
		Tf1 (R)	GCTTTWADKGAATSBCCGCC		
Surfactin	*srf*A-A	As1 (F)	CGCGGMTACCGVATYGAGC	419–431	Tapi et al., 2010
		Ts2 (R)	ATBCCTTTBTWDGAATGTCCGCC		
Bacillomycin	*bmy*B	BmyB (F)	GAATCCCGTTGTTCTCCAAA	370	Mora et al., 2011
		BmyB (R)	GCGGGTATTGAATGCTTGTT		
Iturin	*itu*D	ItuD (F)	TTGAAYGTCAGYGCSCCTTT	482	Chung et al., 2008
		ItuD (R)	TGCGMAAATAATGGSGTCGT		


### Physicochemical Characterisation of Biosurfactant

The L193 purified biosurfactant was characterised as explained above using the oil-spreading test (Morikawa et al., 2000), the emulsification index (Cooper and Goldenberg, 1987) and by measuring surface tension reduction and critical micelle concentration (CMC) (Phale et al., 1995). Finally, the chemical composition of the surfactant was determined by ultra performance liquid chromatography time-of-flight mass spectrometry (UPLC-TOF MS).

To determine the CMC, the reduction in surface tension in a 0.2% (w/v) surfactant solution and its serial dilutions were measured at 25°C using a Kruss K11 tensiometer. The CMC of the L193 biosurfactant corresponded to the highest dilution of the surfactant that produced the maximum reduction in surface tension.

The chemical composition of the biosurfactant was analysed by UPLC-TOF MS using an Acquity UPLC^®^ BEH300 C4 column (1.7 μm, 2.1 × 50 mm). The mobile phase used was a mixture of acetonitrile and water buffered with a solution containing 0.1% (v/v) formic acid in water. Analysis conditions were as follows: 10 μL of sample at 40°C and an analysis time of 12 min.

Mass spectrometry analysis was carried out by positive electrospray ionisation (ESI+) using a MS Waters Synapt-G2 device. The analysis was done under the following conditions: capillary 3 Kv, source temperature 100°C, desolvation temperature 500°C, cone gas flow 40 L/h, desolvation gas flow 800 L/h, mass interval of detection from 100 to 2,400 Da and survey scan time 0.1 s. The data obtained were processed with the aid of MassLynx^TM^ software (Waters).

### Insecticidal Activity Bioassay

In order to determine the insecticidal potential of strain L193, the bacterial culture, supernatant and purified biosurfactant were tested *in vivo* against *R. padi* aphids (Agrobio S.L.).

To obtain the different treatments, strain L193 was cultured in TSB medium supplemented with 1% (w/v) colloidal chitin at 28°C in a shaking incubator at 130 rpm for 5 days. The supernatant was obtained by centrifugation at 13,000 rpm for 20 min and was then filtred through a 0.22-μm pore filtre. The biosurfactant was extracted from the 250 mL culture supernatant as described above and was dissolved in 250 mL of sterile water.

Imidacloprid (50 μg/mL) was used as positive control. As negative controls, sterile cell-free TSB medium with 1% (w/v) colloidal chitin was used for comparison with the whole culture and the supernatant treatments, while sterile water was used for comparison with the biosurfactant treatment.

The experimental model consisted of sixty individually grown barley (*Hordeum vulgare*) seedlings per treatment and control. Plants were grown in 0.2 pots filled with artificial soil at 23°C. Three-day barley seedlings (plant height: 6–7 cm) were initially infested with three 3^rd^-instar *R*. *padi* aphids and grown for 7 days in a growth chamber at 25°C, 60% humidity and for a 16 h light/8 h dark photoperiod. To avoid aphid dispersion, each plant was covered with transparent plastic film.

The insecticidal experiment lasted 5 days after the 7 days of plant growth. The treatments and controls were sprayed (0.5 mL per plant) on day 1 and 3 of the assay while ensuring that the whole aphid population and plant surface were covered with the spray solution. The dead and living aphids of each plant were counted daily.

Aphid mortality was calculated using Abbot’s formula (Abbott, 1925), which compares living aphids in each treatment (At) with living aphids in each control (Ac):

$$Mortality(\%)=(1-\frac{At}{Ac})\times 100.$$

### Determination of Aphid Feeding

To test whether the treatments affected aphid feeding capacity, ten barley seedlings infested each with three 3^rd^-instar aphids were used for each treatment and control. The experiment conditions were the same as described above, but in this case 90-mm diameter filtre paper discs were placed at the base of each plant stem to collect honeydew drops from the aphids (Kim and Jander, 2007).

In order to avoid contamination of the filtre paper disc by the soil, a Petri dish (with an opening for the stem) was placed between the filtre and the soil. Likewise, when the treatments and controls were sprayed on the seedlings, the filtre and dish were removed to avoid their contamination. Discs containing honeydew from the aphids were collected after 96 h and were immersed in a 0.1% (w/v) ninhydrin solution in acetone. Discs were oven-dried at 65°C for 30 min and cut into strips. The purple/pink spots were extracted with 4 mL of 90% (v/v) methanol by vortexing for 1 h at maximum speed. The tubes were then centrifuged at 5,000 rpm for 10 min, and supernatant absorbance was measured at 500 nm using 90% (v/v) methanol as blank (Nisbet et al., 1994).

### Microscopy Analysis

After the *in vivo* assay described above, dead aphids from plants treated with the bacterial culture or the biosurfactant, as well as water-control aphids, were collected in order to analyse the effect of treatments using optical, scanning electron and transmission electron microscopy. The samples were prepared as described by Kim et al. (2011). Briefly, for scanning electron microscopy analysis, aphid samples were fixed with 2% (v/v) glutaraldehyde and 2% (v/v) paraformaldehyde in 50 mM cacodylate buffer at pH 7.4 for 48 h at room temperature. After fixing, the samples were washed three times in the cacodylate buffer and dehydrated through 50, 70, 90, and 100% ethanol for 5 min in each stage. The samples were dried and examined with a scanning electron microscope. For transmission electron microscopy analyses, aphid samples were immersed in 2.5% (v/v) glutaraldehyde in 0.1 M PBS buffer at pH 7.4 for 48 h at 4°C. Following washing with the PBS buffer, the samples were immersed in a mixture of 2% osmium tetroxide and 3%(v/v) ferrocyanide (1:1, v/v) in the same buffer for 1 h at 4°C. After a washing with the PBS buffer, the samples were dehydrated in a series of ethanol solutions and embedded in Epon 812. Ultrathin sections were mounted on Formvar-coated nickel grids (200 mesh) and stained with aqueous uranyl acetate and alkaline lead citrate for 5 min. The samples were then examined with a transmission electron microscope.

### Statistical Analyses

Data normality was checked using the Shapiro-Wilk test. Since the data did not follow a normal distribution pattern, even after natural logarithmic and Box-Cox transformation, Kruskal-Wallis and Mood’s median tests were used to determine whether the five treatments differed significantly (*p* ≤ 0.05) in terms of aphid mortality. The Mann-Whitney U test with the Bonferroni correction was then used for pairwise comparisons of the treatments.

## Results

### Microorganism Isolation and Identification

The strain used in this study was isolated from a soil sample taken from the Malahá salt works (Granada) in the south-east of Spain. The 16S rRNA gene sequence (1,355 bp) indicated that the strain belongs to the genus *Bacillus*. It showed 16S rRNA gene-sequence similarity of 99.5% to its closest relative *B. atrophaeus*. The isolate was named *B. atrophaeus* L193.

### Biosurfactant Production

Different culture media were screened in order to determine in which of these media the biosurfactant production was higher (Table 2). TSB supplemented with colloidal chitin, in which strain L193 produced 2.04 g/L of biosurfactants, was selected and used as the culture medium to perform the subsequent experiments.

**Table 2:** Surfactant production by *B. atrophaeus* L193 in different culture media.

	LB	LB chitin	TSB	TSB chitin	Cooper chitin	Cooper glucose	Cooper glucose chitin
Surfactant (mg/L)	502.4	281.2	1620.8	2040.0	203.6	271.6	284.0
Biomass (mg/L)	1640	1424	1908	1840	1516	416	1450
Yield	0.31	0.20	0.85	1.11	0.13	0.65	0.20


### Biosurfactant Detection in L193 Culture on TSB Chitin Medium

**FIGURE 1:**
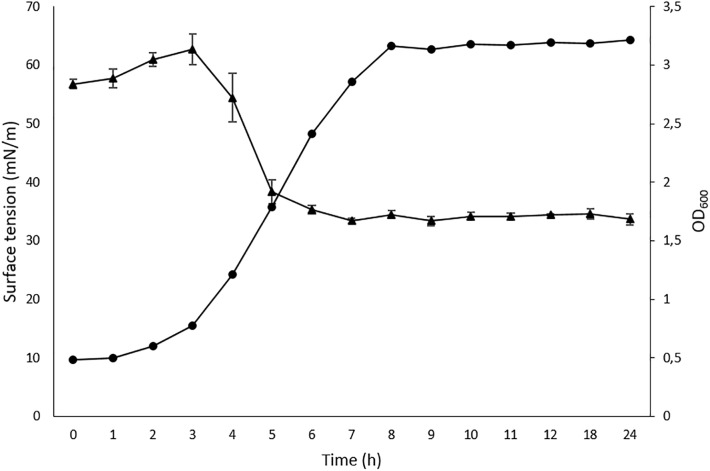
Time-course cell density (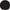) and surface tension (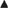) values for *Bacillus atrophaeus* L193. Data represent mean ± standard deviation of the triplicates.

The cell-free supernatant of strain L193 grown in TSB medium supplemented with 1% (w/v) colloidal chitin produced a clear halo zone of 24.95 cm^2^ in the oil displacement test, while the drop-collapsing test was also positive (data not shown). The emulsification index (E_24_) for cell-free supernatant activity against mineral oil, almond oil and isopropyl myristate was 8.08% (±1.24), 42.63% (±2.63), and 3.73% (±1.31), respectively. The reduction in surface tension reached a maximum (33.0 mN/m) after 4.5 h of incubation (Figure 1).

### Determination of Hydrolytic Enzyme Production

In this study, we analysed the production of glucosidase enzymes (amylase and chitinase) and proteases (caseinase) by strain L193 in order to determine its potential insecticidal capacity. The undiluted L193 culture supernatants on 1% (w/v) TSB chitin-containing media displayed enhanced amylase, chitinase and caseinase production (Table 3).

**Table 3:** Amylase, chitinase and caseinase activities of *B. atrophaeus* L193 supernatants on TSB medium supplemented with 1% (w/v) colloidal chitin.

	Amylase^∗^	Chitinase	Caseinase
Supernatant (100%)	15	3	10
Supernatant (50%)	14	2	7
Supernatant (25%)	11	1	4


*^∗^Enzimatic activity was determined by measuring the hydrolysis halo (mm) produced in the different culture media.*

### Genetic Characterisation of Biosurfactant

Genomic analysis indicates that *B. atrophaeus* L193 contains non-ribosomal lipopeptide synthetase gene clusters, which include genes involved in producing fengycin, surfactin, bacillomycin and iturin, such as *fenC*, *srf*A-A, *bmy*B and *itu*D (Supplementary Figure S1).

### Physicochemical Characterisation of Biosurfactant

In order to analyse the efficiency of biosurfactants extracted from strain L193, critical micelle concentration (CMC) as well as emulsification and oil spreading (displacement) activity were measured. The value obtained for CMC was 9.38 mg/L. The oil spreading test showed a displacement halo area of 33.87 cm^2^ for L193 relative to 55.42 cm^2^ for the positive control Triton X-100 (Supplementary Figure S2). The emulsification index (E_24_) for L193 biosurfactant activity against mineral oil, almond oil and isopropyl myristate was 51.53% (±1.39), 46.87% (±1.46), and 38.05% (±11.21), respectively (Supplementary Table S1). These emulsification indices for the surfactant produced by L193 were slightly lower than those for the positive control Triton X-100. Except with regard to almond oil, all the values for the biosurfactant were higher than those for the supernatant of L193 cultures. The emulsification index of water (negative control) for these compounds was 0% (Supplementary Figure S3).

**FIGURE 2:**
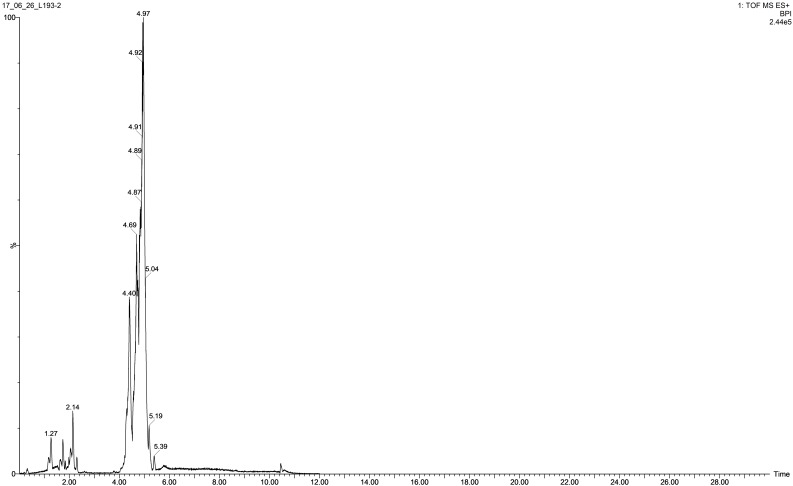
TIC spectrum obtained from the surfactant of *B. atrophaeus* L193 cultured in TSB medium supplemented with 1% (w/v) colloidal chitin.

Ultra-performance liquid chromatography time-of-flight mass spectrometry (UPLC-TOFS MS) was used to identify metabolites produced by the isolate tested. Figure 2 illustrates the total ion chromatogram (TIC) spectrum of the *B. atrophaeus* L193 culture extract.

**FIGURE 3:**
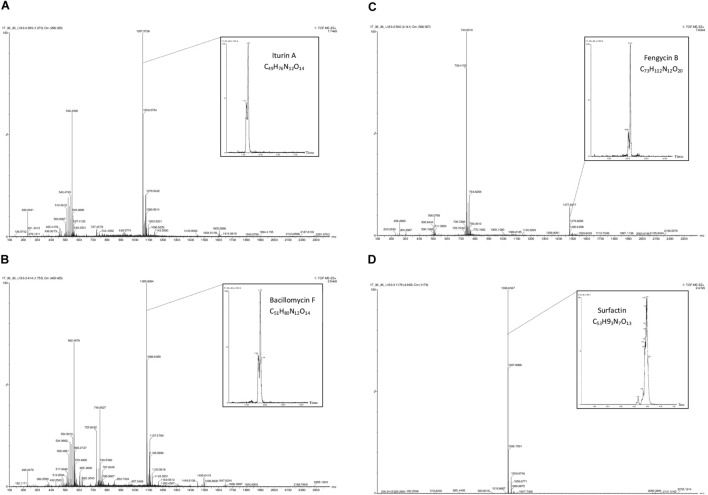
Q-TOF MS spectra obtained from the surfactant produced by *B. atrophaeus* L193: protonated linear derivatives of the [M+H] of iturin A **(A)**, bacillomycin F **(B)**, fengycin B **(C)**, and surfactin **(D)**.

The analyses show that strain L193 can produce various types of lipopeptides. Four known surfactins, with an acyl chain ranging from C12 to C15, as well as two known types of bacillomycin F (C15 and C16) were detected. Iturin A and fengycin B peaks were also observed (Table 4). UPLC-TOF MS detected two predominant compounds: an [M+H] peak at m/z 1,057.5736 for iturin A with molecular formula C_49_H_76_N_12_O_14_ and an [M+H] peak at m/z 1,036.6947 for surfactin with molecular formula C_53_H_93_N_7_O_13_ (Figures 3A,D, respectively). Also an [M+H] peak at m/z 1,085.6064, for bacillomycin F with a molecular formula C_51_H_80_N_12_O_14_ and an [M+H] peak at m/z 1,477.8317 for fengycin B, with a molecular formula C_73_H_112_N_12_O_20_ (Figures 3B,C, respectively) were detected. No mass signals were assigned to other lipopeptides. This agrees with the results obtained by the PCR analysis (Supplementary Figure S1).

**Table 4:** Lipopeptide production by *B. atrophaeus* L193 as detected by Q-TOF MS.

Lipopeptide	Fatty chain length	[M+H]	Retention time (min)	Area (%)	Peak intensity
Iturin A	C15	1057.5736	1.27	2.00	1.95e4
Bacillomycin F	C15	1071.5880	1.53	0.37	4.77e3
	C16	1085.6064	1.74	1.18	1.86e4
Fengycin B	C15	1477.8317	2.15	2.54	5.99e3
Surfactin	C12	994.6420	4.21	0.05	9.92e3
	C13	1008.6595	4.40	15.47	9.55e4
	C14	1022.6754	4.69	2.31	1.29e5
	C15	1036.6947	4.97	72.50	2.46e5


### Insecticidal Activity Bioassay

**FIGURE 4:**
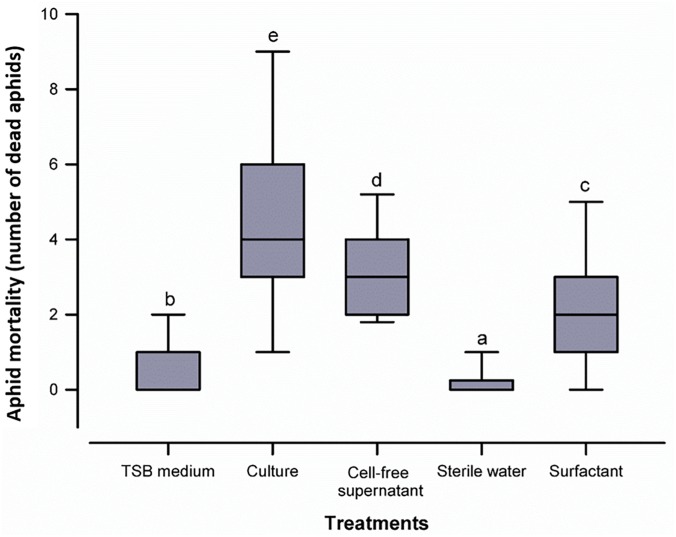
Box plot representing aphid mortality with the five treatments tested. The centre line of the box depicts the median, the edges of the box reflect the 25th and 75th percentiles (interquartile range), and the whiskers depict the 10th and 90th percentiles. Different letters above the whiskers indicate significant differences (*p* ≤ 0.05) between treatments according to the Mann-Whitney U test.

In order to evaluate the aphicidal activity of *B. atrophaeus* L193, *in vivo* studies were conducted in barley plants infested with three 3^rd^-instar *R*. *padi* aphids, and treated or untreated with strain L193 culture, cell-free supernatant or purified extract. The results show high levels of activity against *R. padi* for all of the treatments. The differences between the treatments and control were, in all cases, highly significant (*p* ≤ 0.05). The highest mortality rate of aphids was obtained 24 h after the first treatment (Figure 4). Imidacloprid, a neonicotinoid commonly employed for aphid control, with 100% mortality after 24 h, was used as positive control (data not shown in Figure 4).

Given the corrected mortality based on Abbot’s formula, an enhanced insecticidal activity was observed in the biosurfactant (59.8%) as compared to the culture (50.6%) and the cell-free supernatant (47.7%) of *B. atrophaeus* L193 (Supplementary Table S2).

### Determination of Aphid Feeding

Aphid feeding capacity was evaluated by measuring the amount of honeydew excreted by the whole aphid population after 96 h. The samples corresponding to the L193 culture and cell-free supernatant treatments showed a reduction of 38.5 and 42.9% in the honeydew excreted, respectively, as compared to controls. L193 surfactant treatment samples showed a reduction of 28.7% as compared to controls.

### Microscopy Analysis

**FIGURE 5:**
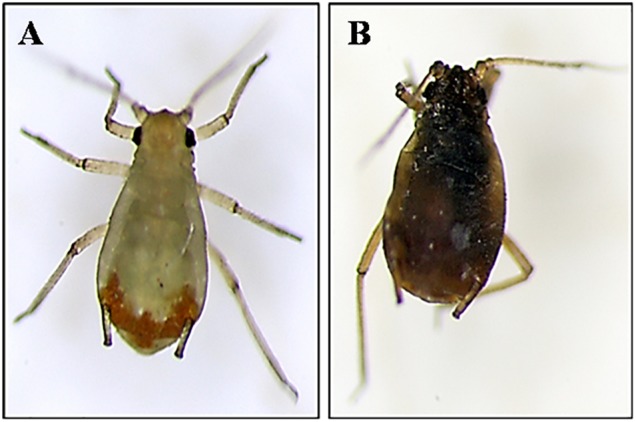
Signs of infection in *Rhopalosiphum padi* caused by *B. atrophaeus* L193 observed by stereo microscopy. **(A)** Treatment with water (control). **(B)** Treatment with L193 surfactant.

The signs of *R. padi* infection caused by topic treatment with *B. atrophaeus* L193 were observed by optical microscopy and are shown in Figure 5. Aphids exposed to L193 ceased to move, their bodies were dehydrated and their green colour darkened.

**FIGURE 6:**
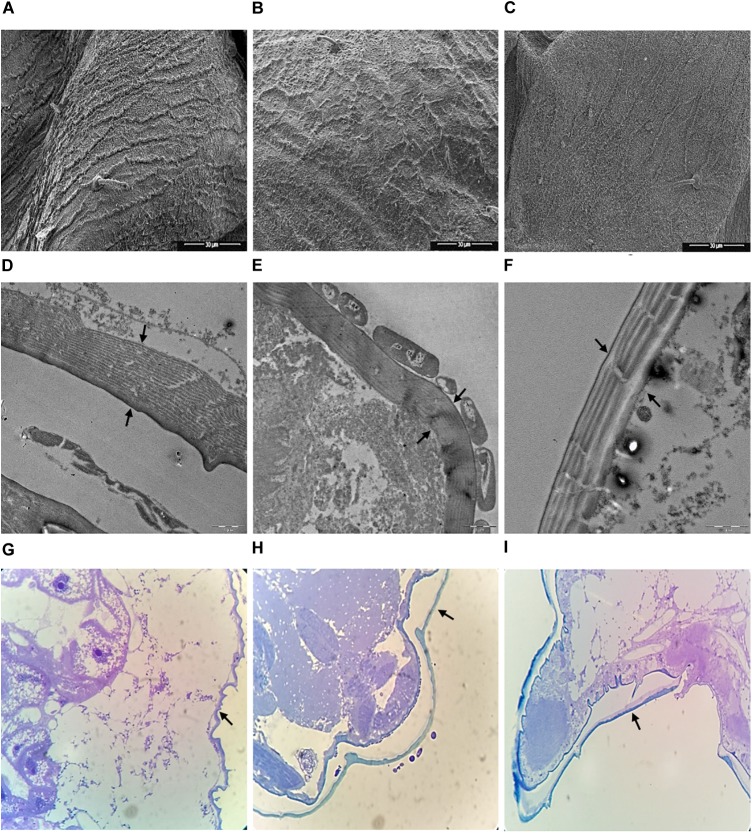
Micrographs of aphid thoraxes produced by scanning electron microscopy **(A–C)**, transmission electron microscopy **(D–F)** and optical microscopy **(G–I)** of aphids treated only with TSB medium **(A**, **D,** and **G)**, the L193 culture **(B**, **E,** and **H)** and the biosurfactant produced by L193 **(C**, **F,** and **I)**. Arrows indicate aphid membranes.

Scanning electron microscopy (SEM) and Transmission electron microscopy (TEM) data on aphids treated with TSB medium and the L193 culture or the biosurfactant produced by L193 are shown in Figure 6. SEM analysis of aphids treated with TSB medium alone showed an intact cuticle (Figure 6A), while clear evidence of damaged cuticle was detected in aphids treated with L193 culture and the biosurfactant (Figures 6B,C, respectively). TEM and optical microscopy confirmed that treatment with strain L193 affects the cuticular membrane of the aphids, with a 36.23% reduction in thickness in the case of the bacterial culture treatment (Figure 6E) and a 58.35% reduction with the L193 surfactant treatment (Figure 6F). With regard to treatment with the biosurfactant, a clear change in cuticle structure was also observed.

With regard to treatment with the L193 culture, TEM micrographs also highlighted the presence of a bacterial cell monolayer firmly attached to the cuticle surface of aphids (Figure 6E), with bacterial cells sometimes appearing to cause the development of a pseudopod-like structure in the aphid cuticle (Supplementary Figure S4A). SEM micrographs showed cuticle concavity in the aphids (Supplementary Figure S4B).

## Discussion

The strain used in this study showed 16S rRNA gene-sequence similarity of 99.5% to its closest relative *B. atrophaeus*. The isolate was named *B. atrophaeus* L193.

Several studies have previously highlighted the potential of different *Bacillus* strains to act as biocontrol agents against insect pests. The effect of these bacteria has been tested in relation to a wide range of agronomically important pests including aphids (Dutton et al., 2003; Palma et al., 2014; Rashid et al., 2017). As explained elsewhere, these insects are considered to be the most destructive agents affecting several crops and the agricultural economy (Leroy et al., 2011).

Most studies have analysed the biopesticidal activity of the genus *Bacillus* against the green peach aphid *Myzus persicae*, one of the most destructive pests, which causes huge crop loss (Torres-Quintero et al., 2015). The most common *Bacillus* species tested against *M. persicae* is the entomopathogenic bacterium *Bacillus thuringiensis* (Torres-Quintero et al., 2015). This bacterium produces crystalline inclusions composed of insecticidal crystal proteins (ICP) and endotoxins. Although these ICPs and toxins are highly active against several orders of insects, various studies have shown low to moderate toxicity to aphids (Porcar et al., 2009). It would therefore be interesting to study the potential of other *Bacillus* species to act as biocontrol agents against these insects. To the best of our knowledge, this is the first study to analyse the anti-aphid insecticidal activity of *B. atrophaeus*, which we tested against *Rhopalosiphum padi* (L.) (*Homoptera*: *Aphididae*), one of the most abundant and economically important aphids in both winter and spring wheat crops.

Biosurfactant production by L193 was tested in different culture media. The data showed that the culture medium used in the growth of the microorganism can decisively influence biosurfactant production. TSB supplemented with colloidal chitin was selected for the experiments.

The use of biosurfactants from microorganisms is a potentially effective pest management strategy. In order to detect biosurfactant production by L193, the oil displacement test, drop-collapsing test and the emulsification index of the cell-free supernatant of strain L193 grown in TSB medium supplemented with colloidal chitin were used. The pattern observed was also found with respect to other biosurfactants produced by microorganisms (Lee et al., 2008).

The efficiency of biosurfactants extracted from strain L193 was evaluated by measuring the critical micelle concentration (CMC) as well as emulsification and oil spreading (displacement) activity. CMC values for the different biosurfactants tested range from 9 mg/L (Sivapathasekaran et al., 2009) to 140 mg/L (Mukherjee and Das, 2005). The low CMC value (9.38 mg/L) for L193 indicates that this biosurfactant is more efficient than other biological surfactants (Sriram et al., 2011).

The oil spreading test and the emulsification indices used for the surfactant produced by L193 showed results very similar to those for positive controls such as Triton X-100. These properties of L193 lipopeptides, together with their CMC, highlight their potential biotechnological, biomedical and environmental applications due to their ability to reduce interfacial tension between aqueous and oleous mixtures.

In this study, we also analysed the production of glucosidase enzymes (amylase and chitinase) and proteases (caseinase) by strain L193 in order to determine its potential insecticidal capacity, as the insect cuticle is composed mainly of chitin nano-fibres embedded in a matrix of proteins, polyphenols and water with small amounts of lipid. Chitin is also a common constituent of insect exoskeletons which support the epidermal cuticles and the peritrophic matrices lining the gut epithelium (Merzendorfer and Zimoch, 2003).

The role of such lytic enzymes in the insecticidal activity of this strain has previously been observed in the genus *Bacillus* (Driss et al., 2011). The undiluted L193 culture supernatants on 1% (w/v) chitin-containing media displayed enhanced chitinase, amylase and caseinase production. Driss et al. (2011) have highlighted the important role played by the chitinase enzyme in *B. thuringiensis* whose integration into ICPs enhanced their insecticidal activity. The synergistic effect of proteases and chitinases on the cuticle degradation mechanism has also been demonstrated (Leger et al., 1986). Moreover, our study demonstrates that L193 produces glucosidases and proteases, thus confirming the insecticidal potential of these enzymes.

Different *Bacillus* sp., which produce lipopeptides with surfactant properties, have been described (Raaijmakers et al., 2010). These metabolites are regarded as the most common class of compounds produced by *Bacillus* sp. (Stachelhaus and Marahiel, 2002). The antifungal activity of lipopeptides isolated from *B. atrophaeus* has also been described (Huang et al., 2015). Genomic analysis carried out in our study indicates that *B. atrophaeus* L193 contains non-ribosomal lipopeptide synthetase gene clusters, which include genes involved in producing fengycin, surfactin, bacillomycin and iturin genes.

Aleti et al. (2016) have reported that the lipopeptides fengycins, iturins and surfactins produced by *B. atrophaeus* 176s display antifungal activity and can protect different crops against *Rhizoctonia solani* infection. However, very few of these compounds produced by *Bacillus* spp. have been reported to be involved in aphid control, and their insecticidal activity against this insect has not been studied to any significant extent.

The metabolites produced by L193 were identified using electrospray quadrupole time-of-flight mass spectrometry (Q-TOFS MS), which show that strain L193 can produce four types of lipopeptides: surfactins, bacillomycin F, iturin A, and fengycin B. All these lipopeptides have been previously observed by PCR. Although some studies have described the control of plant pathogens by surfactins (Chen et al., 2013), little research has been carried out on the aphicidal activity of these molecules produced by the genus *Bacillus*. To our knowledge, only two studies have analysed the role of surfactins produced by different *Bacillus* sp. acting as aphicidal metabolites (Yun et al., 2013; Yang et al., 2017), while the aphicidal role played by other lipopeptides, such as iturin, bacillomycin and fengycin, remains unclear.

*In vivo* studies of barley plants infested with three 3^rd^-instar *R*. *padi* aphids and treated with *B. atrophaeus* L193. The results show high levels of activity against *R. padi* for all of the treatments tested, L193 culture, cell-free supernatant and biosurfactant.

The highest mortality rate was observed after 24 h of incubation. As indicated above, the predominant metabolites present in the biosurfactant produced by strain L193 were lipopeptides, whose insecticidal activity against *R. padi* was also demonstrated. The mechanisms involved in the anti-aphid activity of lipopeptides produced by the genus *Bacillus* sp. remain unclear. Yun et al. (2013) identified a surfactin acting as an aphicidal metabolite produced by *B. amyloliquefaciens* G1. More recently, Yang et al. (2017) showed that *B. subtilis* Y9 produces biosurfactants which act as insecticidal metabolites against this aphid species. Both studies investigated aphid mortality rates using a topic assay method similar to that used in our study. However, most studies report that the anti-aphid insecticidal activity of the genus *B. thuringiensis* involves ingestion (Bravo et al., 2007).

Aphid feeding was evaluated by measuring the amount of honeydew excreted by the whole aphid population. The samples of L193 culture, cell-free supernatant and surfactant treatments showed a reduction in the honeydew excreted as compared to controls. These results indicate that L193 topic treatments not only affected cuticle integrity but also aphid feeding capacity. Similar results were found in a study of *Bacillus velezensis* YC7010 as an inductor of systemic anti-aphid resistance by plants (Rashid et al., 2017), although, in this case, the treatment was by ingestion.

Microscopy data on aphids treated with the L193 culture or the biosurfactant produced by L193 are given in this study. Scanning electron microscopy (SEM) analysis shows clear evidence of damage to the cuticle in aphids treated with L193 culture and the biosurfactant. Transmission electron microscopy (TEM) and optical microscopy confirmed that treatment with L193 affects the cuticular membrane. With regard to treatment with the biosurfactant, a clear change in cuticle structure was also observed. Lipopeptides appear to induce aphid cuticle dehydration due to interaction with cuticle molecules such as phospholipids and fatty acids (Puterka et al., 2003; Jang et al., 2013). We hypothesise that the biosurfactant produced by L193 affects cuticle lipids and leads to membrane perturbation. This mechanism is corroborated by microscopy observations which showed that the treatment tested in this study causes clear changes in the endocuticle layer of aphids. Similar results have been reported for other biosurfactants, such as rhamnolipids, produced by different species of *Pseudomonas* sp. (Kim et al., 2011; Jang et al., 2013).

*Bacillus atrophaeus* L193 produces a biosurfactant with enhanced physicochemical properties as compared to other biological surfactants described to date. In addition, genomic analysis indicated that L193 contains gene clusters for the biosynthesis of non-ribosomal lipopeptide synthetases. This was confirmed by Q-TOF MS, which detected the presence of different types of lipopeptides in the biosurfactant. The *in vivo* studies carried out suggest that the biosurfactants produced by *B. atrophaeus* L193 may be useful for controlling aphids. To the best of our knowledge, this is the first study to confirm the insecticidal activity of *B. atrophaeus* against *R. padi*, one of the most abundant and economically important aphids affecting both winter and spring wheat crops.

## Author Contributions

MR assisted with experimental techniques and statistical analysis. AM and MT assisted with experimental techniques related to previous screening and taxonomic identification. VB assisted with analysing the results and critical revision of the manuscript. MC designed the insecticidal assay, assisted with analysing the results, and critical revision of the manuscript. IS designed the experimental techniques, analysed the results, and drafted the manuscript.

## Conflict of Interest Statement

The authors declare that the research was conducted in the absence of any commercial or financial relationships that could be construed as a potential conflict of interest.
