# Supplementary material for: Aphicidal Activity of Surfactants Produced by Bacillus atrophaeus L193
Source: Front Microbiol. 2018 Dec 18;9:3114. doi: 10.3389/fmicb.2018.03114 (PMC6305586; doi:10.3389/fmicb.2018.03114)
Supplement: Supplementary file 1 [file Data_Sheet_1.docx]

**Figure S1:** Molecular identification of non-ribosomal lipopeptide synthetases of *Bacillus atrophaeus* L193. Lane 1: molecular weight markers 1000 bp DNA ladder; lane 2: negative control; lanes 3, 4, 5 and 6: fengycin, surfactin, bacillomycin and iturin synthetases genes *fenC*, *srf*A-A, *bmy*B and *itu*D, respectively.

**Figure S2:** Oil spreading test of the *Bacillus atrophaeus* L193 surfactant (A) and Triton X-100 (B) as positive control tested on plated with crude oil.


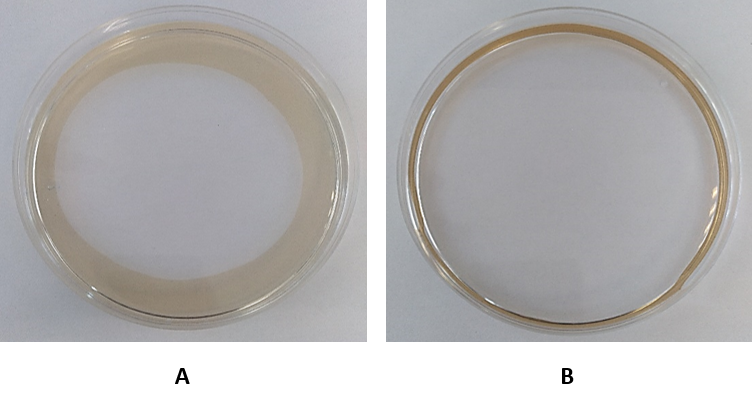


**Figure S3:** Emulsification index (E_24_) of *Bacillus atrophaeus* L193 cell-free supernatant, L193 surfactant and Triton X-100 (positive control) for different hydrophobic compounds. Data represent mean ± standard deviation of the triplicates.


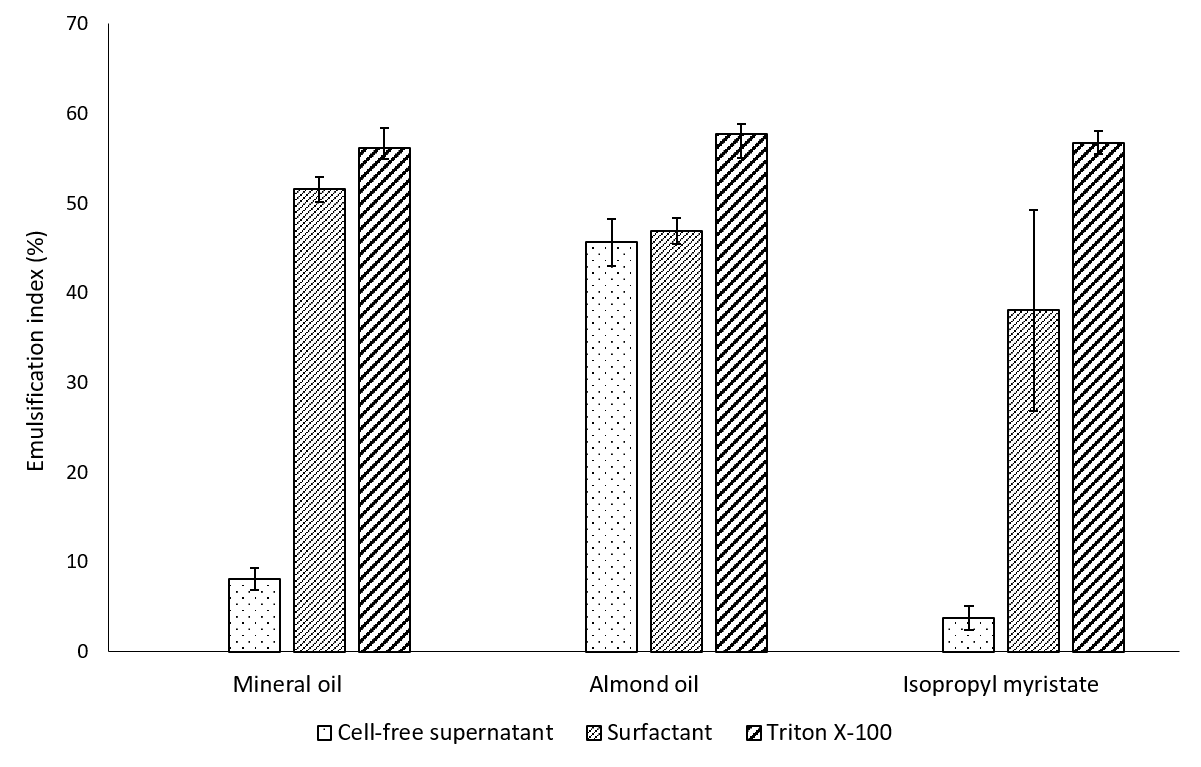


**Figure S4:** Micrographs of transmission electron microscopy (A) and scanning electron microscopy (B) of the aphids treated with the *Bacillus atrophaeus* L193 culture.


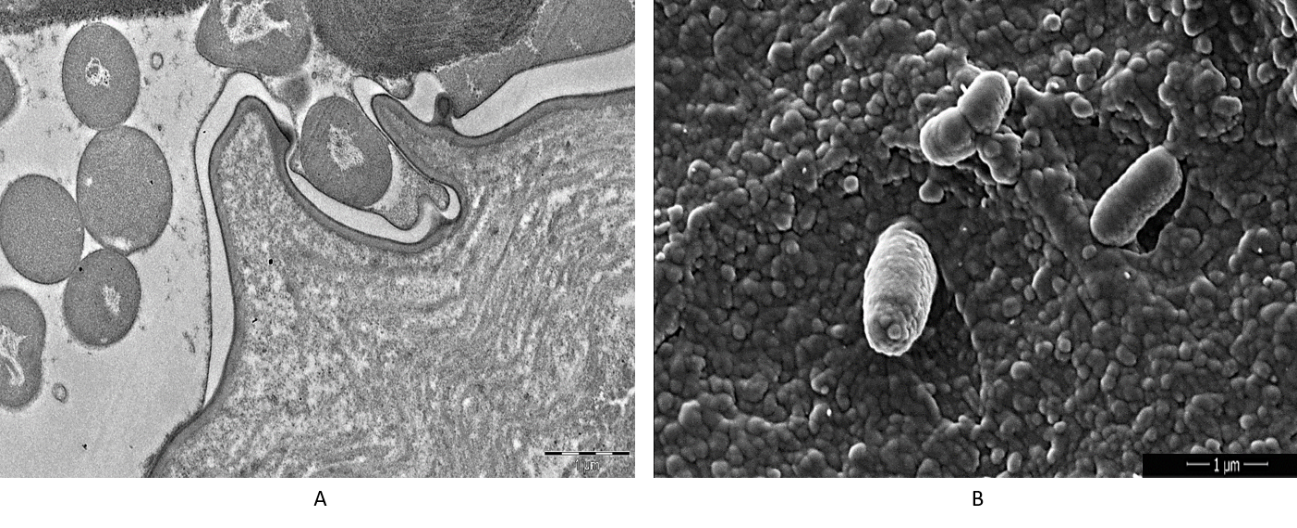


**Table S1:** Physicochemical characteristics of *Bacillus atrophaeus* L193 surfactant in comparison with Triton X-100, a commercial control.

| **Physicochemical characteristics** | **L193** | **Triton X-100** |
| --- | --- | --- |
| Oil spreading test (cm^2^) | 33.87 | 55.42 |
| Emulsification index (% ± SD): |  |  |
| Mineral oil | 51.53 ± 1.39 | 56.19 ± 2.21 |
| Almond oil | 46.87 ± 1.46 | 57.67 ± 1.09 |
| Isopropyl myristate | 38.05 ± 11.21 | 56.74 ± 1.30 |
| Surface tension reduction (mN/m) | 33.0 | 30.0 |
| Critical micelle concentration (mg/L) | 9.38 | 129.4 |

**Table S2:** Mortality percentage according to Abbot’s formula.

| **Treatment** | **Alive aphid** | **Mortality (%)** |
| --- | --- | --- |
| TSB medium | 1222 | 0.0 |
| Culture | 604 | 50.6 |
| Cell-free supernatant | 639 | 47.7 |
| Sterile water | 1345 | 0.0 |
| Biosurfactant | 541 | 59.8 |
